# Complete Genome Sequence of Two Middelburg Viruses Isolated from Arthropods in the Central African Republic

**DOI:** 10.1128/genomeA.01078-14

**Published:** 2014-10-23

**Authors:** Vianney Tricou, Nicolas Berthet, Stéphane Descorps-Declere, Emmanuel Nakouné, Mirdad Kazanji

**Affiliations:** a Department of Virology, Institut Pasteur de Bangui, Bangui, Central African Republic; b Epidemiology and Physiopathology of Oncogenic Viruses, Institut Pasteur, Paris, France; c UMR3569, Centre National de la Recherche Scientifique, Paris, France; d Plateforme de Bioanalyse Génomique, Génopole, Institut Pasteur, Paris, France

## Abstract

Arboviral diseases are a major threat to human and animal health today. Analysis of whole-genome sequences of decades-old arboviral strains may bring new insights into the viral evolution that might have facilitated outbreaks. Here, we report the whole-genome sequences of two Middelburg viruses isolated several decades ago in the Central African Republic.

## GENOME ANNOUNCEMENT

Middelburg virus (MIDV) is an arthropod-borne virus that belongs to the genus *Alphavirus* and family *Togaviridae*. Its single-stranded positive-sense RNA genome contains 2 open reading frames (ORFs) flanked by 2 untranslated regions (UTR), as well as a 5′ cap and a 3′ poly(A) tail. The 1st ORF encodes the nonstructural proteins NSP1 to NSP4. A leaky stop codon near the *nsP3* gene 3′ end allows 2 different polyproteins to be produced. The 2nd ORF encodes the structural proteins, including the capsid protein and the envelope proteins E1 and p62 that later undergo proteolytic maturation into E2 and E3 (1). The MIDV prototype strain Ar-749 was isolated from *Aedes caballus* in South Africa in 1957 (2). The other known vectors include other *Aedes* species and *Mansonia africana* (3). The known vertebrate hosts are sheep, goats, and horses, in which it can cause severe diseases, including encephalitis (4). There is no report of diseases in humans caused by this pathogen. Its geographic distribution includes South Africa, Zimbabwe, Cameroon, Kenya, Senegal, and the Central African Republic (CAR) (3). Based on their serological cross-reactivities, alphaviruses can be classified into antigenic complexes. MIDV is the only representative of the MIDV complex. Based on molecular phylogeny, MIDV has been placed either within or below the Semliki Forest virus complex (5, 6). Only 1 whole-genome sequence is available in GenBank corresponding to MIDV-857, isolated from a horse in Zimbabwe (4). Here, we report the whole-genome sequences of MIDV ArB-8422 and ArTB-5290 isolated in 1977 from *Aedes vittatus* and in 1984 from *Amblyomma variegatum*, respectively, during arthropod surveillance in CAR. These were amplified by serial passage in the brains of newborn mice. After the 3rd passage, the brains were homogenized in Hanks’ balanced salt solution and centrifuged, and the supernatants were lyophilized. These lyophilizates were resuspended in sterile water only recently. RNA was extracted using the QIAmp viral RNA minikit (Qiagen) and then treated with DNase to remove contaminating DNA; it was retrotranscribed using SuperScript III enzyme and random hexamers (Life Technologies). Amplification was done using the Phi29 enzyme, as described previously (7). Sequencing was performed by GATC Biotech (Konstanz, Germany) using the HiSeq 2000 sequencer (Illumina). Whole-genome sequences were assembled as one contig using SPAdes version 3.0.0 (8). The overall lengths are 11,468 and 11,550 nucleotides, and the average coverages are 908× and 1,559× for ArTB-5290 and ArB-8422, respectively. The coding sequence lengths are 7,236 and 3,777 nucleotides for the 1st and 2nd ORFs, respectively. Both viruses share >98% nucleic acid identity with the MIDV-857 virus. The foremost difference with MIDV-857 concerns the 3′ UTR that exhibits different duplication patterns, with ArTB-5290 and ArB-8422 having an absence of duplications present either in the MIDV857 or Ar-749 strains. The 3′ UTR of alphaviruses is known to contain direct repeats, but their role is still unclear even if it has been hypothesized that they have beneficial effects on the adaptation to vectors or hosts (9, 10). Further studies of these sequences would help to better understand the relationships among alphaviruses and to anticipate the emergence of new arboviral diseases.

### Nucleotide sequence accession numbers.

The whole-genome sequences are available in the DDBJ/EMBL/GenBank database under accession numbers KM115530 and KM115531.
